# Dynamics of the water-plasma interface in various discharge modes of atmospheric-pressure plasmas

**DOI:** 10.1038/s41598-026-45989-x

**Published:** 2026-04-01

**Authors:** Aiymgul Toremurat, Azamat Ashirbek, Ainur Akildinova, Yerbolat Ussenov, Sagi Orazbayev, Merlan Dosbolayev, Tlekkabul Ramazanov

**Affiliations:** 1 Institute of Applied Sciences and Information Technologies, 280 Bayzakov Str., Almaty, 050040 Kazakhstan; 2 NNLOT, Al-Farabi Kazakh National University, 71 Al-Farabi Ave., Almaty, 050040 Kazakhstan; 3 Princeton Plasma Physics Laboratory, Princeton, NJ 08543 USA; 4 Institute of Experimental and Theoretical Physics, Al-Farabi Kazakh National University, 71 Al-Farabi Ave., Almaty, 050040 Kazakhstan; 5 Kazakh Physical Society, 71 Al-Farabi Ave., Almaty, 050040 Kazakhstan

**Keywords:** Atmospheric pressure plasma, Plasma-liquid interactions, Streamer discharge, Electrohydrodynamic forces, Engineering, Physics

## Abstract

This paper presents an experimental study of the dynamics of the water-plasma interface during the interaction high-voltage (HV) atmospheric pressure discharge with the water surface. Under the influence of the voltage applied to the pin type electrode, discharges are formed at the air layer between the sharp-tip HV electrode and deionized water. Three discharge regimes were identified from synchronized electrical waveforms and imaging: (i) a weak linear regime at 3–10.6 kV, (ii) a branching streamer regime at the maximum applied voltage of 12.6 kV, and (iii) a continuous arc-like regime occurring during the transition to lower voltage and higher current (e.g., U ≈ 3.2 kV, I ≈ 4.07 mA). High-speed shadowgraph images showed a symmetric interfacial cavity whose depth increased nonlinearly with voltage from h_0_ ≈ 0.1 mm at 3 kV to h_0_ ≈ 2.7 mm at 10.6 kV, reaching a maximum depth of h_0_ ≈ 5.9 mm at 12.6 kV, while the cavity disappeared in the continuous-channel regime and was replaced by outward-propagating wave-like motion. The deformation of the water surface during the discharge-water interaction is governed by the balance between electric field forces, surface tension, gravitational forces, and electrohydrodynamic forces, whose relative contributions vary with the applied voltage. This effect was explained by quantitatively estimating the magnitudes of the acting forces and establishing the force balance. Optical emission spectroscopy in the continuous-channel regime indicated air plasma signatures and yielded a gas temperature of approximately  400 K, while prolonged operation (≈ 20 min) increased the water temperature to ~ 70 °C, reduced the water-layer thickness from 6 mm to 3 mm, and decreased pH from 7 to 4. These regime-resolved, quantitative results clarify how the dominant interfacial forcing shifts with discharge mode and provide a mechanistic basis for controlling plasma-water interactions in atmospheric-pressure applications.

## Introduction

Discharges at atmospheric pressure interacting with liquid electrodes have become a significant subject of research in modern low temperature plasma science and technology. Plasma-activated water (PAW) or plasma-treated liquids (PTL) are used in medicine for wound healing, infection prevention, and water disinfection^1–5^, as well as in agriculture to enhance seed germination^6–10^. These technologies are effective for environmental remediation^11,12^, water purification^6,13,14^, production of green fertilizers^15^, and are also promising tools for the synthesis of nanomaterials and polymers^5,16^.

Studies in^17–19^ have shown that in systems with liquid electrodes, various streamer structures - thin, branching, or stable plasma channels - can form depending on the dynamics of voltage and current variations. In^20,21^, the stability of the liquid surface and structural changes at the liquid-plasma interface were experimentally described, particularly surface deformations and interface instabilities accompanied by increased current. A study^22^ showed that the interaction of a weakly ionized helium plasma jet with the water surface leads to stabilization of the cavitation cavity due to an electrohydrodynamically induced gas flow (electric wind), suppressing the development of Rayleigh and Kelvin-Helmholtz type instabilities. Historically, most studies in this field are based on plasma jets interacting with liquid surfaces or on the generation of (PAW).

The interaction of gas-flow-driven plasma jets with liquid interfaces has already been extensively studied by several groups^22–25^. Although gas-flow-induced plasma jets enable stable plasma formation and are widely employed in practical applications such as cold plasma surface treatment, the study of a pure plasma discharge column and its self-induced gas flow remains valuable for understanding the fundamental plasma-driven processes underlying liquid surface deformation, instability formation, and cavitation at the liquid electrode interface. P. Bruggeman et al.^26^ found that for small inter-electrode distances the water surface instability, taking a Taylor cone-like shape, was necessary to trigger the electrical breakdown. At the moment of breakdown, the water elevation was considerably higher than the maximum stable height observed during static elevation measurements. For larger gaps (7 mm and greater), breakdown occurred when the water surface remained static, as the voltage threshold was below the surface stability limit. Y. Zhang et al.^27^ observed that the negative DC corona discharge generated an ionic wind that caused a localized depression or “hollow” on the water surface, and they calculated the wind pressure based on the depth and radius of this deformation. They determined that an optimal gap distance of 3 mm maximized both the resulting wind pressure (up to 33 Pa) and the active area of the depression. Furthermore, the study noted a self-rotation of the corona discharge, which caused the center of the depression to trace a circular pattern, effectively expanding the area uniformly treated by the ionic wind. N. Zehtabiyan-Rezaie et al.^28^ conducted a numerical investigation showing that the depth of the air-water interface deformation increased as the ionic force and impinging ionic wind strengthened (higher electric Reynolds numbers). This deformation was particularly significant in shallow water layers, potentially reaching 20% of the initial water depth. The simulation confirmed that the interface deformation was primarily driven by the EHD flow pressure rather than the direct electrical surface pressure attracting the water surface. A. Dickenson et al.^29^ concluded that the dominant mechanism driving liquid flow and surface impact correlates with the liquid’s charge relaxation time. For highly conductive tap water, the liquid behaved as a conductor, leading to stable, minimal surface deformation driven predominantly by the EHD flow induced in the gas phase. In contrast, for low-conductivity de-ionized water, electric surface stresses dominated the liquid flow, causing large, unstable surface deformations.

The aim of this study is to conduct a comprehensive investigation of discharge dynamics during its interaction with distilled water at atmospheric pressure, as well as to analyze water surface deformation through force balance analysis during the interaction process at different gas discharge propagation phases in the absence of directed gas flow. The central novelty of our study is that we investigate water-plasma interface dynamics in a direct-discharge configuration (high-voltage needle above distilled water) without any externally imposed gas flow, specifically to isolate discharge-driven interfacial phenomena from flow-fed jet effects. While gas-flow plasma jets are highly practical and well-studied, the absence of directed flow in our setup makes it possible to examine how the discharge itself (electric-field forcing, momentum transfer, and plasma-induced hydrodynamics) governs surface deformation and discharge evolution. This addresses a gap in the literature: a single, unified experimental picture that connects discharge regime evolution, electrical signatures, and water-surface deformation, and then explains the observed interface response with a consistent quantitative force-balance argument across regimes.

This work considers a configuration in which the high-voltage electrode is located directly above the liquid surface, allowing the discharge to interact directly with the liquid without the on additional gas flow. Such systems facilitate the study of direct plasma-liquid interactions, bypassing the complexities of gas-phase processes. This approach is particularly relevant for applications requiring the localised treatment of liquid media. The discharge behavior during plasma-water interaction was classified into three distinct regimes on the basis of synchronized voltage - current measurements and imaging, and the corresponding interface response was tracked (cavity formation and nonlinear deepening, maximum deformation, and subsequent disappearance with wave-like motion). Beyond qualitative description, the magnitudes of electrostatic pressure, surface tension, gravitational, and electrohydrodynamic forces were estimated and compared, demonstrating how the dominant contributions change with operating conditions and how this governs the onset and evolution of the observed cavities. The interpretation of discharge-regime transitions was additionally supported by optical emission spectroscopy and accompanying thermal/physicochemical observations, linking the electrical behavior and plasma state to the shift from electrostatic-cavity-dominated behavior to thermal/EHD-dominated hydrodynamic motion.

The findings significantly contribute to a deeper understanding of the fundamental processes governing plasma-liquid interactions, which is crucial for optimizing applications in water purification, biomedical engineering, and materials processing.

## Experimental setup and methods

**Fig. 1:**
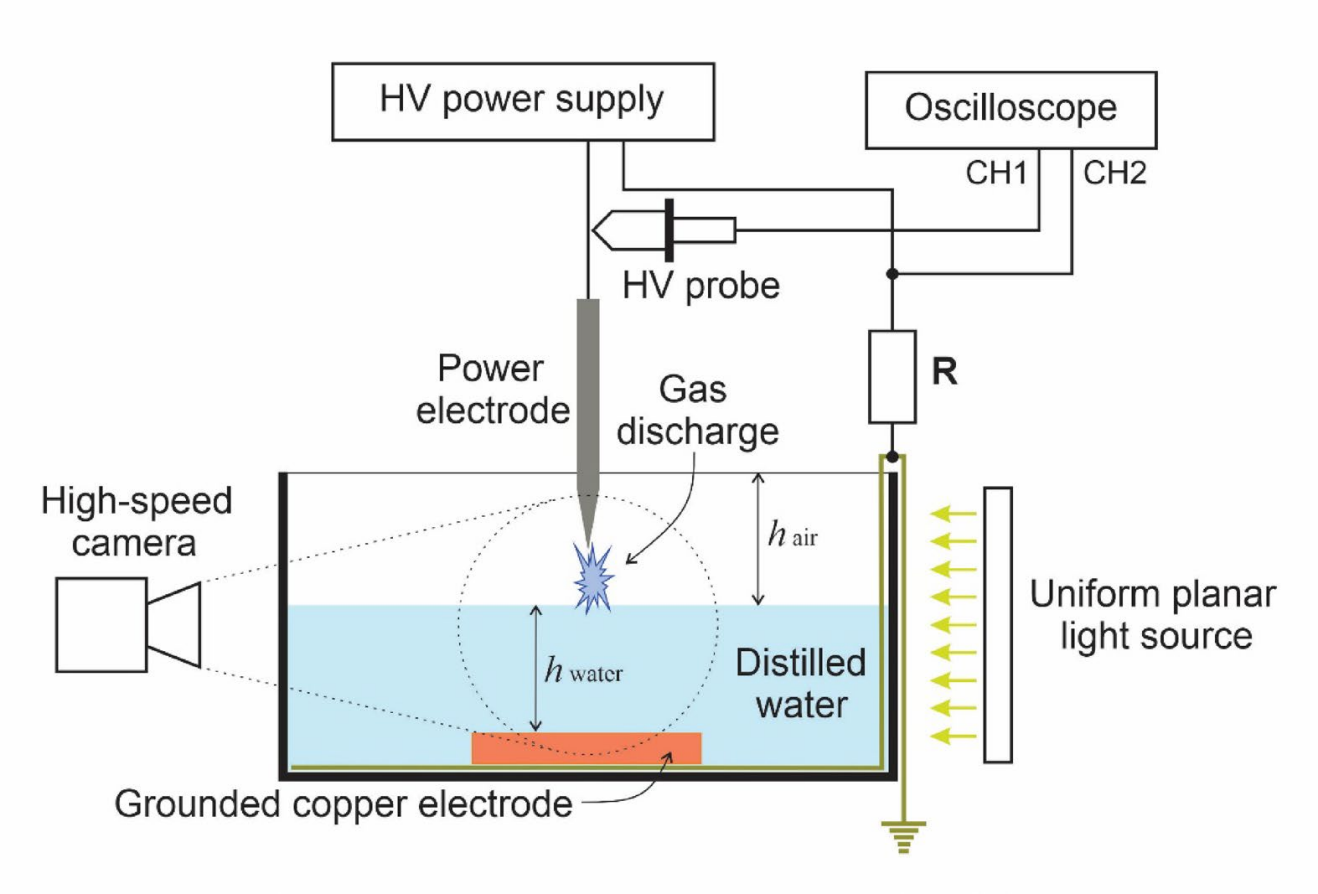
Schematic of the experimental setup.

This section describes the experimental setup and methodology used to study the dynamics of discharge at the gas discharge-water interface. Figure 1 shows a schematic diagram of the experimental setup. The gas discharge is ignited between the tip of the high-voltage electrode and the surface of distilled water under the action of a sinusoidal high-voltage source with a fixed frequency of 27 kHz.

The high-voltage electrode represents a rod made of AISI 304 stainless steel with a sharpened tip, having a length of $\:{l}_{e}=120\:mm$ and a diameter of $\:{d}_{e}=3.75\:mm$, while the radius of curvature of the tip is $\:{r}_{tip}\approx\:0.27\:mm$. The air gap between the electrode tip and the water surface is $\:{h}_{air}=9\:mm$. The container, with dimensions of $\:45\times\:49\times\:45{\hspace{0.17em}}{\mathrm{mm}}^{3}$, is made of acrylic and is equipped with a side quartz window with a diameter of 30 mm. A grounded copper disk with a thickness of 2.9 mm and a diameter of 25 mm is installed at the bottom of the container and serves as the counter-electrode. Distilled water at room temperature with an electrical conductivity of 18.2 µS/cm was used in the experiment. The container was filled so that the thickness (height) of the water layer was 6 mm, measured from the surface of the copper electrode to the water surface.

During the experiments, the water was exposed to a gas discharge for durations of up to approximately 20 min. A high-voltage sinusoidal AC power supply (PVM-500, USA) was connected to the high-voltage electrode. The electrode voltage was measured using a Tektronix P6015A high-voltage probe with an attenuation ratio of 1:1000. The current was measured in accordance with Ohm’s law. For this purpose, a low-inductance shunt resistor with a constant resistance of $\:R=57{\Omega\:}$ and a rated power dissipation of 50 W was used. The voltage across the resistor terminals was proportional to the current flowing through it. Voltage and current signals were recorded using a SIGLENT SDS 1204X-E oscilloscope with a bandwidth of 200 MHz.

To visualize the discharge images and the processes occurring on and beneath the water surface during the experiment, the shadowgraph technique was employed: a light source (MegaLight 100, Mitutoyo Corporation, Japan) was directed toward the water surface, and a high-speed camera (Phantom VEO710S) was positioned on the opposite side. The image sequences were recorded at a selected sampling rate of 40,000 f/s with an exposure time of 24.6 µs, and for different applied voltage values, representative frames corresponding to the moments when the cavity depth was most pronounced were extracted from the recorded sequences for further analysis and presentation. For additional visual monitoring, static images of the discharge were also captured using a smartphone camera. These images represent time-integrated light emission accumulated over multiple AC voltage cycles. Therefore, they are intended for qualitative visualization of discharge regimes and overall streamer morphology, rather than for quantitative analysis of instantaneous streamer dynamics or branching.

The plasma was diagnosed using broadband optical emission spectroscopy (OES). The light emitted from the discharge was collected using a fiber-optic cable installed perpendicular to it. The optical emission spectra were recorded using an ATP2000 spectrometer (Opto-Tech) operating in the spectral range of 200–1100 nm with a spectral resolution of 0.5–4.0 nm, depending on slit width. Gas temperature was determined by fitting the prominent spectral peaks using MassivOES^30^.

To monitor water temperature during the experiment, a thermal probe from the multifunctional Quality Water Meter device (Multiparameter Meter EC900, Biobase Biolin Co., Ltd., China) was placed in the water, providing continuous measurement throughout the experiment. To assess changes in the physicochemical properties of the water, the pH level was measured before and after the experiment. Changes in the thickness of the water layer were also recorded. These parameters enable quantitative evaluation of the effects at the plasma-liquid interface.

## Results

Based on the results of the study, the discharge behavior during plasma - water interaction was classified into three stages. These stages were differentiated according to the voltage applied to the high-voltage (HV) electrode and corresponding discharge current.

**Fig. 2:**
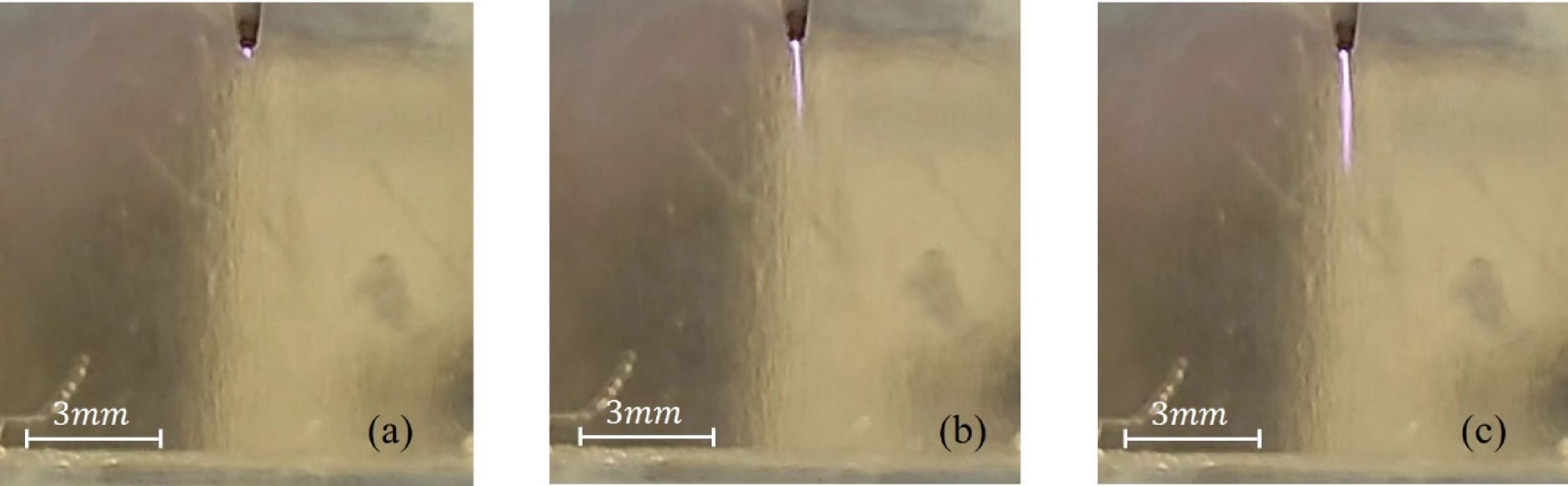
Time-integrated discharge images captured using a digital camera at different applied voltages $\:(\mathrm{U}=3-10.6\:\mathrm{k}\mathrm{V})$: (**a**) initial discharge (≈3 kV); (**b**), (**с**) forming streamer channel (Interval 3–10. 6 kV). The images were obtained using a long exposure time and therefore represent the cumulative light emission over multiple AC cycles.

**Fig. 3:**
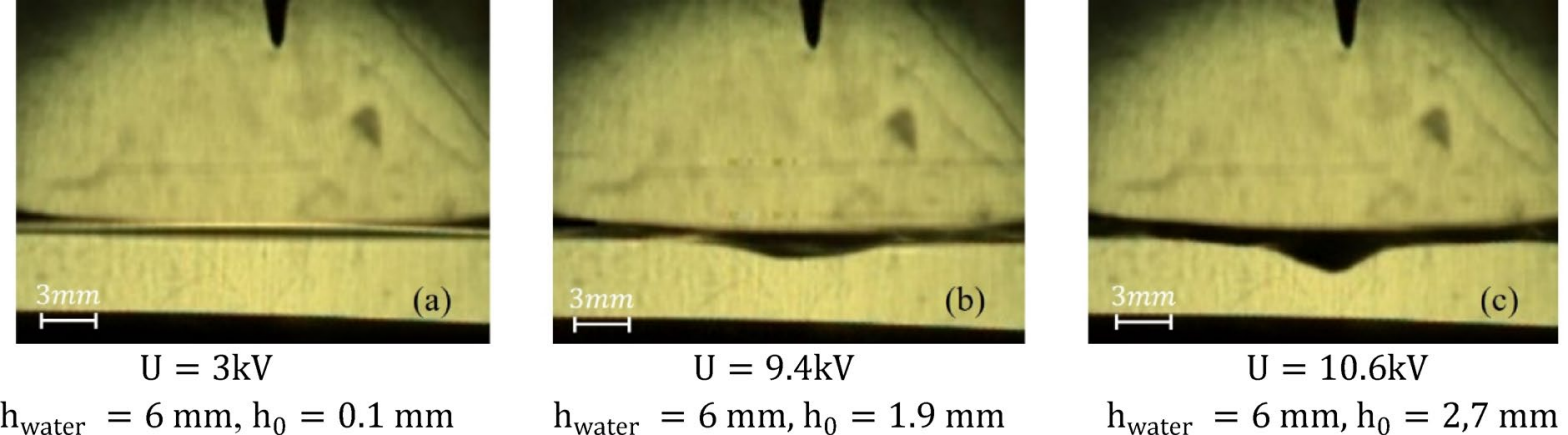
Symmetric hydrodynamic cavity formed at the liquid interface (stage 1). The image was extracted from a high-speed video recorded at a sample rate of 40,000 f/s with an exposure time of 24.6 µs.

The first stage corresponds to the voltage range of 0–10.6 kV. At the initial stage of gradual voltage increase (within the range of 0–3 kV), no distinct discharges were observed; only weak wave-like oscillations were detected on the water surface. Further, as the voltage was gradually increased from 3 kV to 10.6 kV, a weak discharge began to form at the tip of the high-voltage electrode, developing slowly and forming a continuous linear structure (Fig. 2a–c), while no characteristic discharge sound was detected. At the discharge location on the water surface, a small cavity appeared, and its depth increased as the voltage rose (Fig. 3a–c); however, this increase was nonlinear.

**Fig. 4:**
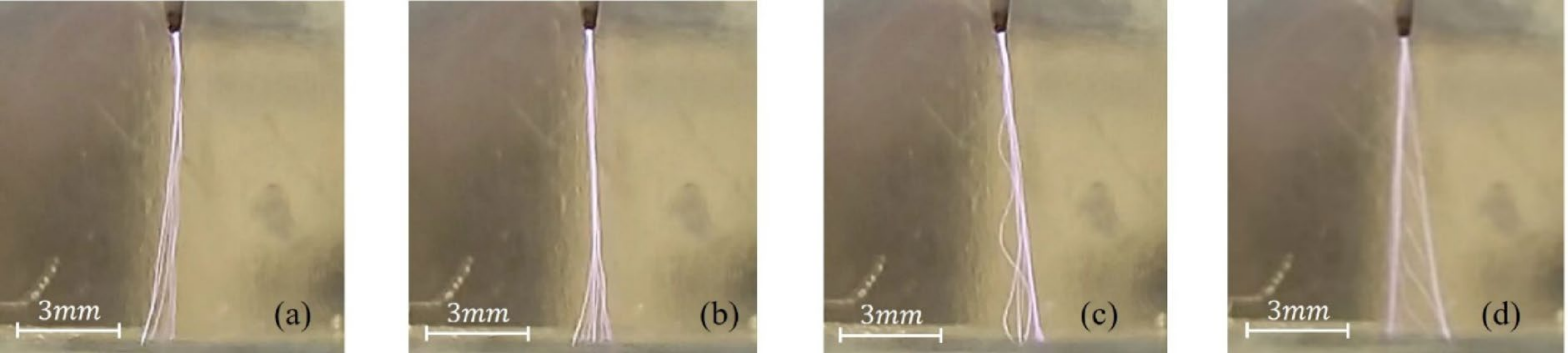
(**a**–**d**) Time-integrated images of streamer structures recorded using a digital camera at $\:\mathrm{U}=12.6\:\mathrm{k}\mathrm{V}$. Due to the long exposure time, the observed branching reflects the superposition of streamer channels formed over multiple AC cycles rather than the instantaneous branching of a single streamer.

**Fig. 5:**
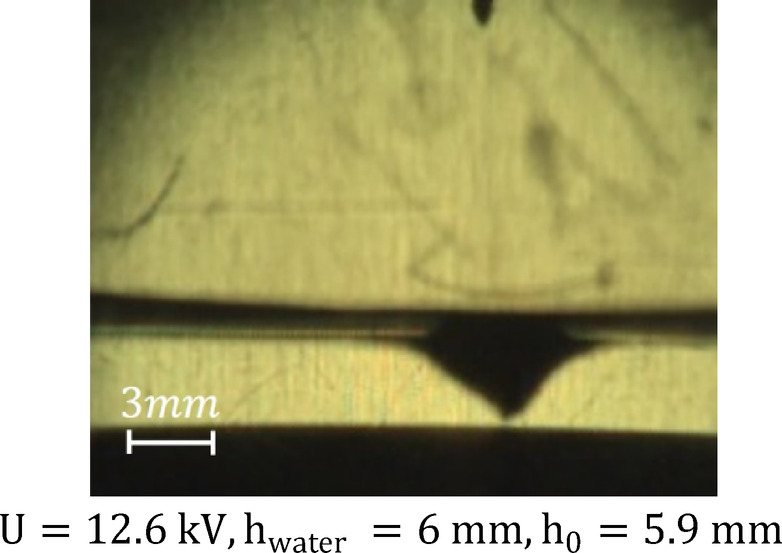
Electrohydrodynamic cavity (stage 2). The image was extracted from a high-speed video recorded at a sample rate of 40,000 f/s with an exposure time of 24.6 µs The frame corresponds to the moment of maximum observed surface deformation.

In the second stage, characterized by the maximum voltage value, the formation of a distinct discharge was accompanied by a sharp sound effect, while the plasma channel branched out in multiple directions, creating complex spatial structures (Fig. 4a–d). At the same time, the depth of the cavitation cavity increased and reached its maximum value (Fig. 5). In the second stage, the signals remained sinusoidal, as in the first stage, differing only in amplitude. Figure 7a shows the voltage and current oscillogram at 9.6 kV.

**Fig. 6:**
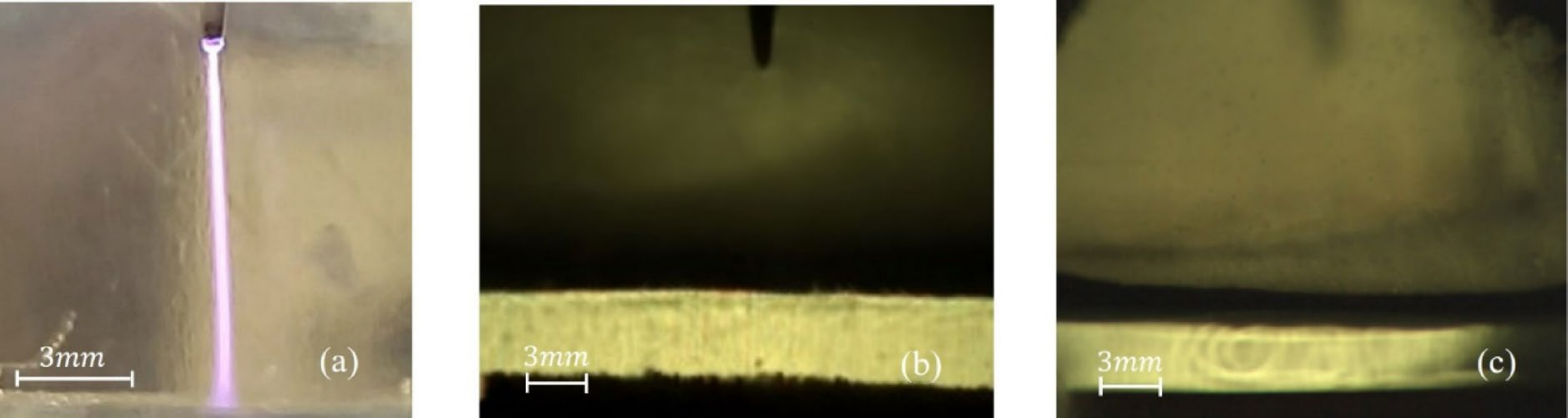
(**a**) Time-integrated images of a continuous discharge channel in stage 3, recorded using a digital camera with long exposure; (**b**) Surface condition of water in the third stage: $\:\mathrm{U}=3.2-4.6\:\mathrm{k}\mathrm{V}$; $\:{\mathrm{h}}_{\mathrm{w}ater}=6\:\mathrm{m}m$, $\:{\mathrm{h}}_{0}=0$. The image was extracted from a high-speed video recorded at a sample rate of 40,000 f/s with an exposure time of 24.6 µs; (**c**) Surface and internal flows induced by thermal plasma channel action.

**Fig. 7:**
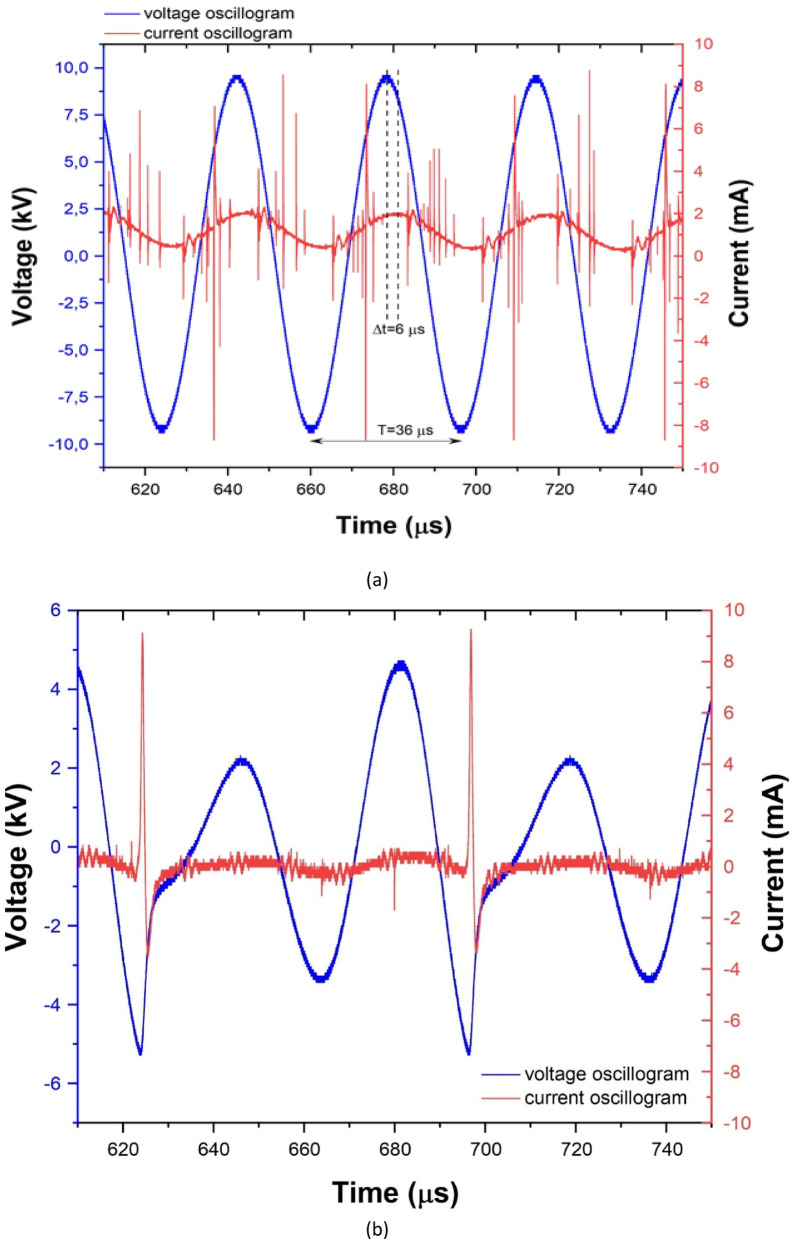
(**a**) Voltage and current waveforms of the atmospheric gas discharge at the gas-liquid interface ($\:U=9.6\:kV,\:f=27\:kHz)$. (**b**) Voltage and current waveforms of the atmospheric gas discharge at the gas-liquid interface in discharge stage 3 recorded at $\:U\approx\:4.6\:kV$ ($\:f=27\:kHz)$. Short current pulses up to ~ 9 mA appear on the capacitive current background.

The experiment revealed that the maximum voltage did not exceed $\:12.6\:\mathrm{k}\mathrm{V}$. With further increases in power supply output, the voltage, conversely, dropped sharply while the current increased significantly: at $\:\mathrm{U}=3.2\:\mathrm{k}\mathrm{V},\:\mathrm{I}=4.07\:\mathrm{m}\mathrm{A}$. This led to a change in the discharge type. The discharge began to propagate with a continuous violet flame-like appearance (Fig. 6a) and, at higher current values, was accompanied by audible clicking sounds, resembling an arc-like discharge. The cavity on the water surface disappeared (Fig. 6b), and instead, symmetrical wave-like motions propagated outward from the discharge region (Fig. 6c). The oscillograms showed an increase in the current amplitude (Fig. 7b). It was clarified that the above phenomena remained unchanged by changing the voltage values ​​​​at the output of the power source in the range of 3.2–4.6 kV.

When the discharge was applied for 20 min in this stage, the water temperature was recorded at 70 ^°^C. The temperature increase led not only to evaporation but also to a significant reduction in water volume: the water layer thickness decreased from 6 mm to 3 mm. This reduction in water layer thickness is directly related to the prolonged operation of the discharge in stage 3.

By the end of the experiment, the pH value had decreased significantly compared to the initial level: while the initial pH was 7, it dropped to 4 by the end of the experiment.

## Discussion

### Hydrodynamic and electrical processes in the first discharge regime (0–10.6 kV)

The difference between the presence of a very weak discharge up to 3 kV and the absence of noticeable changes in the water surface means that the electric field is not sufficient for the formation of a discharge at the distance between the electrode and the water surface^31^. When the voltage reaches 3 kV, the electric field increases and ionization begins near the electrode, which leads to the formation of a discharge (Fig. 2a). As the voltage is further gradually increased up to 10.6 kV, the electric field intensifies and the discharge becomes more pronounced (Fig. 2b,c).

After the discharge occurs, accelerated charged particles formed near the tip of the electrode reach the surface of the water and transmit their momentum to neutral molecules. As a result, a downward pressure force is formed at the plasma-liquid interface due to electrohydrodynamic effects. The surface tension of the water counteracts this pressure. However, when the force generated by the plasma exceeds the total contribution of surface tension and hydrostatic pressure, the water surface deforms inward to form a symmetrical cavitation cavity (Fig. 3a–c). The depth of the cavity increases non-linearly with increasing voltage, which indicates a complex relationship between the electric field strength and the surface tension of water. According to^26,32^, the effect of an electric field on a liquid is determined both by processes in the near-surface region at the molecular level^32^ and by local field amplification due to the geometry of the electrode and edge effects^26^.

To describe the mechanical deformation of the water surface during discharge-liquid interaction, the contributions of the following forces were considered: electrostatic force, surface tension force, gravitational force and electrohydrodynamic (EHD) force. The quantitative values of each of these forces were calculated using the following formulas:

1. Following classical electrohydrodynamic theory, the action of the electric field on the liquid surface can be described in terms of the Maxwell electrostatic pressure^33^.
  1 $$\:{\mathrm{F}}_{\mathrm{e}}=\frac{1}{2}{{\upepsilon\:}}_{0}{{\upepsilon\:}}_{\mathrm{r}}{\mathrm{E}}^{2}{\uppi\:}{\mathrm{r}}^{2}$$

where $\:{{\upepsilon\:}}_{0}=8.85\times{10}^{-12}\:F/m$ - is the vacuum permittivity, $\:{{\upepsilon\:}}_{\mathrm{r}}$ - is the relative permittivity of water, $\:\mathrm{E}$ - is the electric field strength, and $\:\mathrm{r}=3\:\mathrm{m}\mathrm{m}$ - is the radius of the cavitation cavity. This force allows for calculating the effect of the electric field generated by the electrode on the water surface. At voltages of 0 and 2 kV, the measured temperature was 23^°^С, at 3, 9.4 and 10.6 kV, the recorded temperatures were 23^°^С, 24.8^°^С and 25.4^°^С, respectively. Based on this, the relative permittivity of distilled water at room temperature (20–25^°^С) was used for calculations: $\:{{\upepsilon\:}}_{\mathrm{r}}\approx\:81$. The electric field strength was calculated for the three specified voltage values, assuming a distance of 9 mm between the tip of the HV electrode and the water surface.

At the initial stage of the first regime (0–2 kV), due to the absence of a cavitation cavity on the water surface, the cavity area was not taken into account in Eq. (1). For voltage values of 3, 9.4, and 10.6 kV, the electrostatic force values were calculated using Eq. (1).

Since the electrostatic force is proportional to the square of the voltage, its contribution to the balance of forces acting on the surface of the water increases dramatically with increasing voltage. This leads to a change in the relationship between the electrostatic force, the force of surface tension and gravity.

2. The local deformation of the water surface can be described in terms of surface tension force^34^:
  2 $$\:{\mathrm{F}}_{{\upsigma\:}}=2{\uppi\:}\mathrm{r}{\upsigma\:},$$

where $\:{\upsigma\:}$ is the surface tension coefficient of water at the air-water interface. According to the recorded temperatures (23^°^С, 24.8^°^С, 25.4^°^С), the corresponding surface tension values are$\:72.282\times{10}^{-3}\frac{N}{m},\:72.001\times{10}^{-3}\frac{N}{m},\:71.907\times{10}^{-3}\frac{N}{m}$, respectively.

Based on the voltage and current data, it can be concluded that an increase in current is accompanied by moderate heat generation, leading to a slight decrease in the coefficient of surface tension of water. However, the contribution of the surface tension force to the overall balance of forces changes significantly less than with a sharp increase in the electrostatic force.

3. The gravitational force acting on the mass of water is given by:
  3 $$\:{\mathrm{F}}_{\mathrm{g}}={\uprho\:}\mathrm{V}\mathrm{g},$$

where $\:\mathrm{V}$ – is the volume of the cavity and $\:{\uprho\:}=1000\:\frac{\mathrm{k}\mathrm{g}}{{\mathrm{m}}^{3}}$ is the density of water. The cavity is assumed to have a conical shape, and the corresponding depth values $\:\mathrm{h}$ are provided in Fig. 3.

4. The electrohydrodynamic force acting on a liquid arises as a result of momentum transfer by charged particles (ions and electrons) generated in a plasma near the plasma-liquid interface^35^. When these particles interact with the surface of the liquid, volumetric forces are formed that contribute to the emergence of electrohydrodynamic flows. According to^36,37^, under discharge conditions on the surface of a liquid, such forces can lead to the formation of symmetrical flow structures. However, in the first discharge mode, characterized by a weak current and an unstable plasma channel, the flow of charged particles is insufficient to form pronounced EHD flows. As a result, the contribution of the EHD force to the deformation of the water surface is insignificant compared to the contribution of the electrostatic force, surface tension force and gravity.

By combining the calculated quantitative values of the forces (Table 1) and the corresponding conclusions, the relationship between the forces in the first stage can be described as follows:

**Table 1:** Calculated values of acting forces in stages 1–3.

Stage	Voltage ($\:\mathrm{U},\:\mathrm{k}\mathrm{V}$)	$\:{T}_{water},\:{}^{0}C$	$\:\sigma\:,\:mN/m$	Electrostatic force ($\:{\mathrm{F}}_{\mathrm{e}},\:{\upmu\:}\mathrm{N}$)	Surface tension force ($\:{\mathrm{F}}_{{\upsigma\:}},\:{\upmu\:}\mathrm{N}$)	Gravitational force ($\:{\mathrm{F}}_{\mathrm{g}},\:{\upmu\:}\mathrm{N}$)	Electrohydrodynamic force ($\:{\mathrm{F}}_{\mathrm{E}HD},\:{\upmu\:}\mathrm{N}$)
1	3	23	72.282	1125.6	1361.8	9.2	3.15
	9.4	24.8	72.001	11050.7	1356.5	175.4	187.5
	10.6	25.4	71.907	14052.2	1354.7	249.3	300.5
2	12.6	35	70.370	18347.6	1325.8	544.7	780.6
3	4.6	70	64.41	2083.43	1213.5	-	-
	3.2	70	64.41	1008			

At

4 $$\:\mathrm{U}=3\:\mathrm{k}\mathrm{V}, \:{\mathrm{F}}_{\mathrm{e}}<{\mathrm{F}}_{{\upsigma\:}}+{\mathrm{F}}_{\mathrm{g}}$$

the electric field is weak, so surface tension dominates and water deformation is negligible. The depressed liquid volume is very small, resulting in a minimal gravitational force;

At

5 $$\:\mathrm{U}=9.4\:\mathrm{k}\mathrm{V}: \:{\mathrm{F}}_{\mathrm{e}}>{\mathrm{F}}_{{\upsigma\:}}+{\mathrm{F}}_{\mathrm{g}}$$

Since $\:{F}_{e}\propto\:{U}^{2}$, the electrostatic force increases significantly and begins to deform the surface. Surface tension weakens as deformation grows, while the gravitational force increases due to cavity deepening;

6 $$\:\mathrm{U}=10.6\:\mathrm{k}\mathrm{V}: \:{\mathrm{F}}_{\mathrm{e}}\gg\:{\mathrm{F}}_{{\upsigma\:}}+{\mathrm{F}}_{\mathrm{g}}$$

the electrostatic force becomes dominant; surface tension slightly decreases with temperature, and the gravitational force increases but remains comparatively smaller.

Water surface deformation occurs only when $\:{\mathrm{F}}_{\mathrm{e}}>{\mathrm{F}}_{{\upsigma\:}}$​, while the shape and stability of the cavitation cavity are constrained by the gravitational force $\:{\mathrm{F}}_{\mathrm{g}}$.

Under conditions where $\:{\mathrm{F}}_{\mathrm{e}}\gg\:{\mathrm{F}}_{{\upsigma\:}}$​, the deformation intensifies. The increase in gravitational force serves as evidence that the water is displaced not upward, but exclusively downward.

### Stage 2

When the voltage reaches 12.6 kV, an intense discharge develops and a clearly defined branched streamer channel forms toward the water surface (Fig. 4a–d). The formation of streamers is also visible from the current shape. According to Fig. 7a), the voltage oscillogram (blue curve) exhibits a nearly sinusoidal waveform with a period of $\:T\approx\:36\mu\:s$, corresponding to the excitation signal frequency. The current oscillogram (red curve), measured in the grounding circuit, shows a pronounced capacitive component, as indicated by a phase shift of $\:{\Delta\:}t\approx\:6\mu\:s$ $\:\left(\phi\:\approx\:{60}^{0}\right)$, where the current leads the voltage. This phase shift indicates that the discharge at the gas-liquid interface has a predominantly capacitive nature, which is typical for atmospheric-pressure plasma discharges. Sharp current pulses are observed on the background of the capacitive current, which correspond to streamer microdischarges developing at the gas-liquid interface. The presence of several pulses within a single half-period indicates the formation of multiple filamentary discharge channels during one voltage cycle.

Each streamer locally enhances the electric field at the plasma-liquid interface, producing repetitive electrostatic pressure pulses on the water surface. With the transfer of momentum from charged particles accelerated in an electric field to neutral molecules, the water surface is deformed, causing electrohydrodynamic (EHD) forces that exert downward pressure on the water surface (Fig. 5) and it acts against surface tension and hydrostatic forces, which leads to the formation and deepening of cavities.

A quantitative comparison of the forces was performed. At this stage, the relative dielectric permittivity of water at 35^°^С was taken as $\:{{\upepsilon\:}}_{\mathrm{r}}\approx\:74.85$, and the electrostatic force $\:{\mathrm{F}}_{\mathrm{e}}$ was calculated according to Eq. (1) for a voltage of $\:12.6\:\mathrm{k}\mathrm{V}$. The gravitational force acting on the cavity volume at a cavity depth of 5.9 mm was determined using Eq. (3) and the surface tension force was calculated from Eq. (2), with a surface tension value of $\:70.370\times{10}^{-3}\frac{\mathrm{N}}{\mathrm{m}}$ at 35^°^С, which tends to flatten the cavity^38^. This effect is confirmed by the calculated results. The contribution of the EHD force is significant in this regime. It is due to changes in the physicochemical properties of the liquid. During the experiment, the pH of the water decreases, as the concentration of ions increases, which leads to an increase in electrical conductivity. As a result, the polarization properties of the liquid change.

The EHD force can be defined as:

7 $$\:{\mathrm{F}}_{\mathrm{E}\mathrm{H}\mathrm{D}}={{\uprho\:}}_{\mathrm{e}}\mathrm{E}\mathrm{V},$$

where $\:{{\uprho\:}}_{\mathrm{e}}\approx\:0.01\:\frac{\mathrm{C}}{{\mathrm{m}}^{3}}$ and $\:\mathrm{V}$ is the volume of the liquid region upon which the EHD force effectively acts. It should be noted that the EHD force is localized and acts mainly in the area located directly under the tip of the electrode, that is, in the volume of the cavitation cavity.

The calculated values of the forces are presented in Table 1, and at this stage, the balance of forces will be as follows:

8 $$\:{\mathrm{F}}_{\mathrm{e}}+{\mathrm{F}}_{\mathrm{E}\mathrm{H}\mathrm{D}}\gg\:{\mathrm{F}}_{{\upsigma\:}}+{\mathrm{F}}_{\mathrm{g}}$$

This indicates that discharge development is governed by the electric field, while the resistive forces from the liquid (surface tension and gravity) only constrain its dynamics.

Streamer branching arises from local electric field enhancement at the discharge front near the electrode tip. Space charge accumulation increases the field strength, generating new ionization fronts and multiple streamer channels that propagate along the field lines^31^.

Thus, the second stage of the discharge is characterized by the development of a branched streamer structure at high voltage and increasing current, which changes the forces acting on the liquid, as well as its physicochemical properties. The observed discharge dynamics indicate an important phase before the stabilization of the streamer channel and the increase in the impact on the water surface.

### Stage 3

In the third stage, the voltage and current oscillograms indicate a transition to a new discharge regime.

The shape of the current oscillogram indicates the presence of two fundamentally different components; therefore, the total measured current can be represented as $\:I\left(t\right)={I}_{cap}\left(t\right)+{I}_{dis}$, where $\:{I}_{cap}\left(t\right)$ is the capacitive current caused by the equivalent capacitance of the system “needle electrode-gas-gap-water-grounded plane electrode”, and $\:{I}_{dis}$ is the pulsed current of the gas discharge (streamer microdischarges). The capacitive component of the current is determined by the derivative of the applied voltage: $\:{I}_{cap}\left(t\right)\approx\:{C}_{eq}\frac{dV\left(t\right)}{dt}$, and appears as a quasi-sinusoidal background signal present in the current oscillogram even in the absence of breakdown. This component reflects the charging and discharging of the distributed capacitances of the air gap, the water layer, and the surrounding dielectric elements of the experimental setup. Against the background of the capacitive current, short sharp current pulses with amplitudes of up to several milliamperes (~ 9 mA) are observed, corresponding to the ignition moments of the gas discharge. Each such pulse is accompanied by a sharp drop in the voltage at the high-voltage electrode, which is associated with a rapid decrease in the impedance of the gap during the formation of a plasma channel and the corresponding voltage drop across the output resistance of the power supply and the connecting lines.The discharge exhibits an intermittent ignition behavior, meaning that pulses do not appear in every half-cycle. This indicates the important role of charge accumulation and relaxation at the gas-liquid interface (memory effect), which modifies the local electric field. Breakdown is governed by the effective electric field at the needle tip $\:{E}_{eff}\left(t\right)={E}_{V}\left(t\right)+{E}_{\sigma\:}\left(t\right)+{E}_{pol}\left(t\right)$, where $\:{E}_{V}\left(t\right)$ - is the contribution of the external electric field, $\:{E}_{\sigma\:}\left(t\right)$ - is the contribution of the surface charge accumulated at the gas-liquid interface, $\:{E}_{pol}\left(t\right)$ - is the contribution of polarization and the finite conductivity of the water medium. After each streamer event, charge remains on the water surface, partially screening the field and temporarily suppressing breakdown. As this charge relaxes, the condition $\:{E}_{eff}\ge\:{E}_{cr}$ is reached again and the discharge reignites. Pulses occur preferentially during the negative half-cycle, where conditions for electron acceleration and streamer development are more favorable. Overall, discharge ignition is determined by the critical effective field rather than a fixed voltage amplitude.

The current waveform also shows bipolar pulses: the main pulse is followed by a weaker pulse of opposite polarity. This behavior is caused by charge redistribution after discharge extinction rather than a second discharge event. At ignition, the gap resistance drops sharply and the equivalent capacitance rapidly recharges, forming a short high-amplitude pulse; after extinction, a compensation current of opposite polarity appears. Thus, the bipolar shape results from the superposition of discharge current and the transient response of the equivalent RLC circuit. With increasing applied voltage, the influence of charge memory decreases, the external field becomes dominant, and the discharge transitions to a regular periodic regime occurring in every cycle of the excitation signal.

Optical emission spectroscopy (OES) was used to diagnose the active plasma region.

**Fig. 8:**
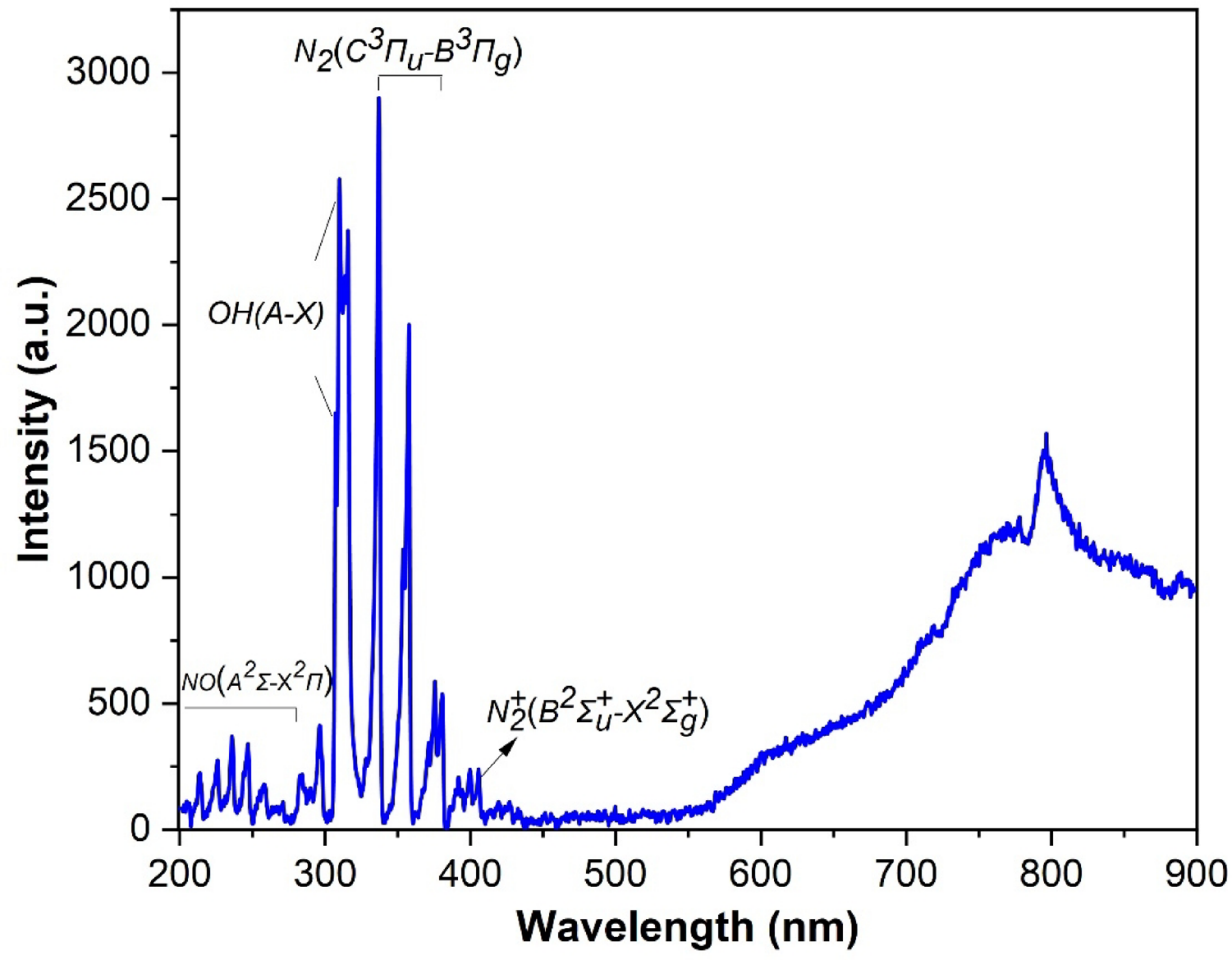
Optical emission spectrum of the discharge in stage 3.

The spectral analysis of the discharge shows several molecular emission bands in the ultraviolet range (Fig. 8). Distinct peaks at approximately 337 nm, 358 nm, and 381 nm correspond to the second positive system of molecular nitrogen $\:\left({N}_{2}\right)$ $\:\left({C}^{3}{{\Pi\:}}_{u}-{B}^{3}{{\Pi\:}}_{g}\right)$. These peaks arise from electronic transitions of the nitrogen molecule and are typical for a gaseous atmospheric plasma, reflecting its molecular spectrum. The weak band around 391–392 nm belongs to the first negative system of ionized nitrogen $\:\left({\mathrm{N}}_{2}^{+}\right)$ $\:\left({\mathrm{B}}^{2}{{\Sigma\:}}_{\mathrm{u}}^{+}-{\mathrm{X}}^{2}{{\Sigma\:}}_{\mathrm{g}}^{+}\right)$. The broadband emission observed in the 500–900 nm range most likely originates from the heated copper electrode rather than from the plasma. In addition, this spectral region contains bands of the $\:{\mathrm{N}}_{2}$ First Positive System $\:\left({\mathrm{B}}^{3}{{\Pi\:}}_{\mathrm{g}}-{\mathrm{A}}^{3}{{\Sigma\:}}_{\mathrm{u}}^{+}\right)$. Additionally, in the 306–309 nm region, the $\:OH\left(A-X\right)$ rotational band is observed, and in the 200–280 nm region, the $\:NO$ vibrational band $\:\left({A}^{2}{\Sigma\:}-{X}^{2}{\Pi\:}\right)$ appears, both of which are characteristic only for plasma in air. Compared to $\:{N}_{2}$ plasma, the emission intensity from the vibrational transitions of $\:{N}_{2}$ $\:\left({B}^{3}{{\Pi\:}}_{g}-{A}^{3}{{\Sigma\:}}_{\mathrm{u}}^{+}\right)$ and $\:{\mathrm{N}}_{2}^{+}$ $\:\left({B}^{2}{{\Sigma\:}}_{\mathrm{u}}^{+}-{\mathrm{X}}^{2}{{\Sigma\:}}_{\mathrm{g}}^{+}\right)$ is lower in air plasma.

The temperatures in the plasma were determined through analysis of the emission spectrum. The gas temperature was determined by fitting the $\:{N}_{2}$ $\:\left({C}^{3}{{\Pi\:}}_{u}-{B}^{3}{{\Pi\:}}_{g}\right)$ bands using MassiveOES and was found to be approximately 400 K, confirming the non-equilibrium nature of the atmospheric plasma despite electrode heating.

The spectral features confirm the presence of atmospheric air plasma in stage 3 and support the electrical observations.

A quantitative evaluation of the forces was also carried out for this stage (Table 1). The electrostatic force $\:{\mathrm{F}}_{\mathrm{e}}$ and the surface tension force $\:{\mathrm{F}}_{{\upsigma\:}}$ were estimated using Eqs. (1) and (2), respectively, with the necessary parameters taken at the appropriate temperature. In this regime, the characteristic radius $\:\mathrm{r}$ represents the effective plasma-liquid interaction area.

In contrast to the previous stages, the disappearance of the cavity (h ≈ 0) makes it impossible to directly evaluate the gravitational and electrohydrodynamic forces using the corresponding expressions, since both depend on the volume of the deformed liquid region. Therefore, these contributions are considered negligible in this regime (the corresponding entries in Table 1 are not defined).

The calculated values ​​show that as the voltage decreases, the electrostatic force becomes approximately equal to the surface tension force $\:\left({F}_{e}\approx\:{F}_{\sigma\:}\right)$, and a further decrease in the electric field leads to $\:{F}_{e}<{F}_{\sigma\:}$ (9). Thus, when the inward electrostatic pressure force exerted by the electric field on the liquid surface^39,40^ is insufficient to overcome the surface tension and gravitational forces, the void disappears and the smooth surface of the water is restored (Fig. 6b).

This indicates a transition from an electrically dominant regime to a regime governed by surface tension.

To provide an intuitive description of the force evolution throughout the discharge stages, the ratio of forces promoting surface deformation $\:\left({\mathrm{F}}_{\mathrm{e}}+{\mathrm{F}}_{\mathrm{E}\mathrm{H}\mathrm{D}}\right)$) to those opposing it $\:\left({\mathrm{F}}_{{\upsigma\:}}+{\mathrm{F}}_{\mathrm{g}}\right)$ was analyzed.

At the beginning of the first stage ($\:3\:\mathrm{k}\mathrm{V}$), this ratio is less than unity, indicating the dominance of surface tension. As the voltage increases ($\:9.4\:\mathrm{k}\mathrm{V}$ and $\:10.6\:\mathrm{k}\mathrm{V}$), the ratio exceeds unity and continues to grow, corresponding to the onset and development of surface deformation.

In the second stage ($\:12.6\:\mathrm{k}\mathrm{V}$), the ratio becomes significantly greater than unity, reflecting the strong dominance of electric forces and the formation of a pronounced cavity.

In the third stage, as the voltage decreases, the ratio returns to values close to unity ($\:4.6\:\mathrm{k}\mathrm{V}$) and subsequently becomes less than one ($\:3.2\:\mathrm{k}\mathrm{V}$). This corresponds to the suppression of surface deformation and the restoration of the initially flat liquid surface.

In Stage 3, besides the diminishing electrostatic forces, thermal effects become significant due to prolonged discharge activity. As a result of the continuous energy, the water temperature in the plasma-liquid interaction zone increases to about 70 °C, which leads to a significant decrease in the surface tension coefficient from $\:\sigma\:\approx\:72\times{10}^{-3}\frac{N}{m}$ at room temperature to $\:\sigma\:\approx\:64\times{10}^{-3}\frac{N}{m}$. This temperature increase leads to the formation of a radial temperature gradient along the liquid surface, with the temperature higher near the discharge zone (70 °C) and decreasing towards the periphery. This difference in temperature on the water surface also leads to a non-uniformity in the surface tension, which creates a surface tension gradient $\:\left(\varDelta\:\sigma\:\right)$. According to the experimental results, the surface tension gradient is $\:\frac{\varDelta\:\sigma\:}{\varDelta\:T}\approx\:0.17\times{10}^{-3}\frac{N}{m}K$. This difference leads to the formation of tangential stresses on the water surface. This causes a tangential force directed from the low (hot) surface tension regions to the high (cold) surface tension regions, which contributes to the redistribution of the liquid surface and affects the boundary flow structure. Under the influence of this driving force, a liquid flow, the so-called Marangoni effect, appears on the surface layer. In the experiment, this effect is confirmed by the spiral movement of water in the discharge ignition zone. As a result, the liquid surface is no longer deformed, but rather a system of surface currents and vortices is formed, which ensure the stability of the self-sustaining plasma channel^41^. The presence of the $\:\frac{\varDelta\:\sigma\:}{\varDelta\:T}$ gradient indicates that the flow field velocity and intense vortex structures appear on the liquid surface, which ensure the stability of the plasma channel. Therefore, at this stage, the system passes into a regime in which thermal effects and Marangoni flows play a dominant role. Along with the weakening of electrostatic forces, thermocapillary and thermal flows control the dynamics of the liquid surface and allow the surface layer to reach a steady state.

## Conclusion

Experimental data showing an increase in cavity depth on the water surface with rising voltage are explained by the strengthening of the electrostatic force due to the increase in the electric field ($\:{F}_{e}\propto\:{U}^{2}$), as confirmed by calculations (stages 1 and 2).

Since the increase in current is accompanied by heat generation and warming of the liuid, the resulting temperature rise leads to a reduction in surface tension, which is confirmed by the calculation results and is consistent with the experimental data (stages 1–3).

The deepening of the cavity in water is also attributed to the water mass, with this contribution being manifested as a relative increase in the effect of gravity. However, as a secondary force, its magnitude negligible and does not exceed that of the surface tension force (stages 1–3). At voltages applied to the high-voltage (HV) electrode in the range of 0–10.6 kV (stage 1), it was established that the electrostatic force becomes dominant, while surface tension weakens slightly, and the gravitational force - although increasing - acts only as a limiting factor for the stability and shape of the cavity, without exceeding the surface tension force. These relationships are described by inequalities (4–6). Additionally, it was determined that surface deformation occurs when $\:{\mathrm{F}}_{\mathrm{e}}>{\mathrm{F}}_{{\upsigma\:}}$​, and increases significantly when $\:{\mathrm{F}}_{\mathrm{e}}\gg\:{\mathrm{F}}_{{\upsigma\:}}$​.

Upon reaching the maximum voltage, the system transitions into a critical regime (stage 2), characterized by the formation of branching streamer channels and intense ionization. This confirms that the discharge development is driven by the electric field, while the opposing forces of the liquid (surface tension and gravity) merely constrain its dynamics (inequality 8).

With increasing current, the discharge transformed into a continuous and stable plasma channel. At this stage, electrohydrodynamic effect became the primary driving forces, inducing vortex structures and wave-like motion on the liquid surface. In this regime (stage 3), thermal and hydrodynamic forces begin to dominate the system, replacing classical electrostatic effects governed by the electric field. This was supported by a comparative analysis of electrostatic, gravitational force magnitudes. Although the numerical values of these forces are close to zero, the weak influence of the electric field still persists; therefore, their relationship is conditionally described by inequality (9).

The results of the study confirmed that the formation and disappearance of cavities on the water surface are governed not only by mechanical equilibrium, but also by a balance of forces that vary depending on the specific discharge regime. The work elucidated the mechanism of stabilized thermal plasma channel formation and the phase-specific hydrodynamic response of the water surface. Understanding these mechanisms holds significant practical potential for the development of plasma-activated water technologies, environmental purification, plasma-based medical treatments, and the synthesis of plasma-derived nanomaterials.

Future work will include experiments with various liquids to investigate the effects of electrical conductivity, surface tension, and viscosity. In addition, different electrode geometries, advanced optical diagnostics, and numerical simulations of plasma-liquid interactions will be considered.

## Data Availability

The data supporting the main findings of this study are included in the paper. Additional data from this study are available from the corresponding author upon reasonable request.
